# MAFLD is linked to lower HBV DNA levels in chronic hepatitis B: clinical and transcriptomic insights

**DOI:** 10.3389/fmed.2026.1926645

**Published:** 2026-09-16

**Authors:** Xiaoyun Gao, Xuan Du

**Affiliations:** 1 Department of Geriatric, The Second Affiliated Hospital of Dalian Medical University, Dalian, China; 2 Department of Gastroenterology, The Second Affiliated Hospital of Dalian Medical University, Dalian, China

**Keywords:** chronic hepatitis B, HBV DNA, MAFLD, metabolic heterogeneity, transcriptomic analysis

## Abstract

**Background:**

Chronic hepatitis B (CHB) frequently coexists with metabolic dysfunction–associated fatty liver disease (MAFLD), yet the impact of MAFLD on hepatitis B virus (HBV) replication remains incompletely understood. We aimed to investigate the association between MAFLD and HBV DNA levels and to provide biological context through integrative transcriptomic analyses.

**Methods:**

We analyzed a large retrospective cohort of patients with CHB and assessed the association between MAFLD and serum HBV DNA levels using linear regression models. HBV DNA was analyzed as a continuous outcome after log10 transformation. To provide molecular context for the clinical findings, weighted gene co-expression network analysis (WGCNA) was performed in an independent HBV liver transcriptomic dataset to identify HBV-associated host co-expression modules. These modules were subsequently characterized in independent liver transcriptomic datasets representing distinct metabolic and histological liver contexts using single-sample gene set enrichment analysis (ssGSEA).

**Results:**

MAFLD was independently associated with lower circulating HBV DNA levels. This association varied across MAFLD phenotypes, with the lowest HBV DNA levels observed in overweight- or obesity-related MAFLD. Transcriptomic analyses identified HBV-associated host co-expression programs linked to lipid and cholesterol metabolism, inflammatory signaling, and antiviral responses. Cross-cohort analyses further showed that selected modules varied across distinct metabolic and fibrotic liver contexts.

**Conclusions:**

MAFLD is associated with reduced HBV DNA levels in patients with CHB, especially in overweight- or obesity-related MAFLD. The transcriptomic findings provide complementary biological context for this clinical association and highlight metabolic and immune pathways that warrant further mechanistic investigation.

## 1 Introduction

Chronic hepatitis B virus (HBV) infection remains a persistent global health problem, even in the era of widespread vaccination. While the number of newly diagnosed infections has been decreasing over the years, the overall burden of the disease has not, principally due to the high number of people already carrying the chronic infection. It is estimated that 296 million people globally are currently infected, and many of these individuals will go on to develop cirrhosis, liver cancer or other serious liver diseases (1). This ensures that HBV remains a major long-term burden on health-care systems.

Meanwhile, the prevalence of metabolic dysfunction-associated fatty liver disease (MAFLD) has been rising (2). This is typically characterised by the presence of liver steatosis in combination with metabolic dysfunction, such as overweight or obesity or type 2 diabetes, and its prevalence has become so high that it is often encountered in clinical practice (3, 4). A recent development has been to recognise that MAFLD is not a single disease. Patients within this category may have vastly different metabolic characteristics, which seems to be reflected in different systemic and liver-specific metabolic effects. We are now seeing more patients with a co-existence of MAFLD and chronic HBV infection in the clinic.

Notably, the effect of hepatic steatosis on HBV replication has been inconsistent. Some have shown a negative effect, while others have not (5–9). This complicates the attribution of viral replication levels to steatosis alone. It may instead be better to consider the impact of the associated metabolic milieu. That is, metabolic context, rather than steatosis, may influence HBV behaviour in the liver.

There is also growing evidence that changes in metabolism can impact several aspects of HBV biology. Lipid modifications, particularly in cellular membranes, are directly related to endoplasmic reticulum (ER) function and stress. Lipid remodeling enzymes, such as lysophosphatidylcholine acyltransferase 3 (LPCAT3), have been shown to play a role in this. Recent studies indicate that when LPCAT3 is inhibited, ER stress is increased and the levels of HBsAg and HBV DNA are elevated (10). This suggests that there may be a link between phospholipid remodeling and HBV replication, but the details are still being worked out.

Based on these findings, we examined whether heterogeneity within MAFLD may help explain differences in HBV replication. Rather than considering MAFLD as a single entity, it could be more informative to examine its subtypes separately, as they reflect distinct metabolic states. Here, we integrated clinical and transcriptomic data to investigate this issue from both clinical and molecular point of view. We grouped patients based on metabolic features and explored their clinical profiles, including virologic features. Moreover, we used network-based analyses of gene expression data to explore associations with HBV DNA levels. We sought to integrate these findings to gain insight into the role of metabolic milieu on HBV replication in the real world.

## 2 Materials and methods

### 2.1 Study design and clinical cohort

This retrospective observational study was conducted at the Second Affiliated Hospital of Dalian Medical University (Dalian, China). We screened HBsAg-positive patients evaluated over an approximately 20-year period and included those with chronic HBV infection who had sufficient virological, metabolic, and imaging data to support MAFLD classification and quantitative assessment of HBV replication.

### 2.2 Eligibility criteria

Inclusion criteria were: (i) documented positivity for HBsAg; (ii) quantitative serum HBV DNA measurements and hepatitis B serologic markers, including HBeAg; (iii) fasting metabolic profiling (glucose and lipid panel) with complete anthropometrics allowing BMI calculation; and (iv) liver imaging adequate to assess hepatic steatosis. We excluded patients with malignant liver disease, other viral hepatitis, and other competing causes of chronic liver injury based on clinical records, laboratory results, and imaging review.

### 2.3 Clinical data collection and laboratory measurements

Demographic variables (age and sex) were extracted from the medical records. Blood samples were obtained after an overnight fast. HBsAg was measured using a fully automated chemiluminescent immunoassay analyzer (Cobas 8000 e801). Serum HBV DNA was quantified using a commercial HBV nucleic acid quantification kit (Sansure Biotech Inc., Hunan, China) on a real-time PCR system (SLAN-96P, Shanghai Hongshi Medical Technology Co., Shanghai, China). Triglycerides, total cholesterol, low-density lipoprotein cholesterol (LDL-C), high-density lipoprotein cholesterol (HDL-C), apolipoprotein A1, apolipoprotein B, alanine aminotransferase (ALT), aspartate aminotransferase (AST), and total bilirubin (TBIL) were measured using an automated biochemical analyzer (Siemens ADVIA 2400).

### 2.4 Assessment of hepatic steatosis

Hepatic steatosis was determined by imaging in routine clinical care, including abdominal ultrasound, computed tomography, magnetic resonance imaging, and liver elastography-based imaging. The distribution of electronically retrievable imaging modalities used to identify hepatic steatosis is provided in Supplementary Table S3. When more than one modality was available, the evaluation closest to the index laboratory measurements was used for phenotyping. Some participants underwent more than one imaging examination; therefore, modality categories were not mutually exclusive.

### 2.5 MAFLD definition and metabolic risk abnormalities

MAFLD was diagnosed based on evidence of hepatic steatosis together with at least one of the following: overweight/obesity, type 2 diabetes mellitus (T2DM), or metabolic dysfunction in lean/normal-weight individuals, in line with international consensus criteria (2). For Asian populations, overweight/obesity was defined as BMI≥23 kg/m^2^ and lean/normal weight as BMI <23 kg/m^2^. T2DM was defined according to widely accepted international diagnostic criteria.

For lean/normal-weight individuals, metabolic dysfunction required at least two of the following metabolic risk abnormalities: (1) waist circumference ≥90 cm in men or ≥80 cm in women; (2) blood pressure ≥130/85 mmHg or use of antihypertensive medication; (3) triglycerides ≥1.70 mmol/L or lipid-lowering therapy; (4) HDL-C < 1.0 mmol/L in men or <1.3 mmol/L in women or lipid-modifying therapy; (5) prediabetes, defined as fasting plasma glucose 5.6–6.9 mmol/L, 2-hour glucose 7.8–11.0 mmol/L, or HbA1c 5.7–6.4%; (6) insulin resistance defined as HOMA-IR ≥2.5; and (7) systemic inflammation, defined as high-sensitivity C-reactive protein >2 mg/L.

For phenotype-specific analyses, mutually exclusive MAFLD subgroups were defined using a hierarchical classification. Patients with T2DM were classified as diabetes-related MAFLD (MAFLD-D) irrespective of BMI. Among patients without T2DM, those meeting the overweight/obesity criterion were classified as overweight/obesity-related MAFLD (MAFLD-B). The remaining lean/normal-weight patients who fulfilled the metabolic dysfunction criteria were classified as lean/normal-weight MAFLD (MAFLD-L). Thus, patients who simultaneously fulfilled the diabetes and overweight/obesity criteria were assigned only to the MAFLD-D group and were not counted in the MAFLD-B group.

### 2.6 Outcomes

The primary outcome was serum HBV DNA levels. Because HBV DNA values were right-skewed, regression analyses used log10-transformed HBV DNA as a continuous outcome. For descriptive analyses, patients were additionally stratified by a prespecified high viral load threshold (HBV DNA ≥1 × 10^6 ^IU/mL), consistent with the baseline tables.

### 2.7 Statistical analysis

Continuous variables were assessed according to their distribution. Approximately normally distributed variables are presented as mean ± standard deviation and were compared using Student's t test for two groups or analysis of variance (ANOVA) for more than two groups. Skewed continuous variables are presented as median (interquartile range) and were compared using the Mann–Whitney U test for two groups or the Kruskal–Wallis test for more than two groups. Categorical variables are presented as number (percentage) and were compared using the chi-square test or Fisher's exact test, as appropriate.

Model 1 was unadjusted. Model 2 was adjusted for age, sex, body mass index, alanine aminotransferase, aspartate aminotransferase, triglycerides, total cholesterol, fasting plasma glucose, and HBeAg status. Model 3 additionally included the FIB-4 index as a non-invasive surrogate of liver fibrosis because direct histological fibrosis staging was unavailable in the clinical cohort. HBeAg status was classified as negative (<1.0) or positive (≥1.0), and adjusted analyses were performed using complete cases. Regression coefficients (*β*) and 95% confidence intervals were estimated using HC3 heteroskedasticity-robust standard errors. Model fit was summarized using R^2^ and adjusted R^2^.

For phenotype-specific analyses, MAFLD-D, MAFLD-B, and MAFLD-L were each compared with the non-MAFLD group using the same covariate-adjusted regression framework. Effect estimates are reported as *β* coefficients with 95% confidence intervals. To account for the three subtype comparisons, *P* values were adjusted using the Benjamini–Hochberg false discovery rate procedure. Given the small number of patients in the MAFLD-L subgroup, estimates for this group were considered exploratory and interpreted cautiously. For comparisons among the four metabolic phenotype groups, *post-hoc* pairwise analyses were performed following a statistically significant overall test. Tukey's honestly significant difference test was used following one-way ANOVA, Dunn's test with Holm correction following the Kruskal–Wallis test, and pairwise Fisher's exact tests with Holm correction for categorical variables.

### 2.8 Public transcriptomic datasets

To explore mechanisms linking MAFLD-related host programs to HBV replication, we analyzed publicly available liver transcriptomic datasets from the Gene Expression Omnibus (GEO). GSE83148 comprised 128 liver samples, including 122 HBV-infected liver tissues and six healthy controls. GSE268360 comprised 18 liver RNA-seq samples, including six normal liver samples and 12 MAFLD samples. GSE240729 comprised 67 histologically characterized MASLD liver samples with varying fibrosis stages. Because these GEO datasets represent independent and unpaired cohorts with different phenotype definitions, the cross-dataset analyses were interpreted as exploratory transcriptomic characterization rather than direct external validation of the clinical association between MAFLD and HBV DNA.

### 2.9 WGCNA, ssGSEA, and enrichment analyses

Weighted gene co-expression network analysis (WGCNA) was performed in GSE83148 to identify host co-expression modules associated with HBV virological status. Genes with zero variance were removed, and the top 50% most variable genes ranked by median absolute deviation were retained, resulting in 10,012 genes for network construction. A signed co-expression network was constructed. Candidate soft-thresholding powers of 1–10 and 12–30 in increments of 2 were evaluated using the scale-free topology fit criterion. A soft-thresholding power of *β* = 28 was selected as the lowest candidate power achieving a scale-free topology fit index of approximately R^2^ = 0.85. Modules were identified using dynamic tree cutting with a minimum module size of 30 and deepSplit = 3, followed by merging of closely related modules at a cut height of 0.25. Module eigengenes were subsequently evaluated in relation to HBV virological strata.

For replication-associated modules, eigengene distributions were compared across HBV DNA strata. Effect sizes for the contrast of >1 × 10^6^ versus undetectable HBV DNA were quantified using Cliff's delta to support module prioritization. For downstream module-signature analyses, genes within each selected module were ranked according to module membership (MM). Genes with MM > 0.80 were retained as highly representative module members. When more than 100 genes satisfied this criterion, the 100 genes with the highest MM values were retained; when ≤100 genes met the criterion, all qualifying genes were retained. These MM-based gene sets were used as representative module signatures.

For downstream module-signature analyses, genes within each selected module were ranked according to module membership (MM). Genes with MM ≥0.80 were retained as highly representative module members. When ≤100 genes satisfied this criterion, all qualifying genes were retained; when more than 100 genes satisfied the criterion, the 100 genes with the highest MM values were retained. These MM-based gene sets were used as representative module signatures.

Functional enrichment analysis of the brown-module signature was performed using Metascape (https://metascape.org/), an online platform for gene annotation and enrichment analysis. Gene Ontology (GO) and Kyoto Encyclopedia of Genes and Genomes (KEGG) terms were evaluated, and representative enriched terms were visualized according to their enrichment significance. To further position the brown module within broader biological programs, ssGSEA scores for selected curated MSigDB pathways were calculated in the GSE83148 cohort and correlated with the brown module eigengene derived from the full WGCNA module using Spearman correlation. *P* values from pathway correlation analyses were adjusted using the Benjamini–Hochberg false discovery rate procedure.

Statistical and bioinformatics analyses were performed using R version 4.5.3. Bioconductor version 3.22 was used for Bioconductor-based transcriptomic analyses.

## 3 Results

### 3.1 Baseline characteristics of HBV patients with and without MAFLD

Baseline characteristics of the study population according to MAFLD status are summarized in Table 1. Among 2,057 patients with chronic HBV infection, 385 (18.7%) met the diagnostic criteria for MAFLD. Patients with MAFLD were characterized by a higher body mass index and a more adverse metabolic profile, including elevated triglyceride levels, compared with those without MAFLD. Liver biochemical parameters showed modest but statistically significant differences between groups, whereas markers of hepatic synthetic function were largely comparable. With respect to virological characteristics, patients with MAFLD exhibited lower serum HBV DNA levels compared with those without MAFLD (Table 1). This difference was evident both in analyses of log10-transformed HBV DNA and in categorical comparisons using HBV DNA ≥1 × 10^6 ^IU/mL as the high viral load threshold.

**Table 1:** Baseline characteristics according to MAFLD status.

Variable	MAFLD (*n* = 385)	Non-MAFLD (*n* = 1,672)	*P* value
Demographic and anthropometric characteristics			
Age, years	52.46 ± 11.35	54.35 ± 12.31	0.004
Body mass index, kg/m^2^	27.39 ± 3.55	24.07 ± 3.43	<0.001
Weight, kg	77.00 (70.00–88.00)	67.00 (60.00–75.00)	<0.001
Height, cm	169.25 ± 8.62	167.35 ± 8.04	<0.001
Liver biochemistry			
ALT, U/L	32.28 (23.03–50.84)	24.12 (16.43–41.09)	<0.001
AST, U/L	25.00 (19.83–36.00)	23.80 (18.70–34.76)	0.049
GGT, U/L	27.00 (17.92–49.00)	20.30 (13.23–39.00)	<0.001
ALP, U/L	69.07 (58.27–86.36)	71.75 (58.00–90.00)	0.250
Albumin, g/L	42.46 ± 5.04	40.23 ± 5.89	<0.001
Prealbumin, mg/L	258.60 ± 69.60	221.75 ± 70.68	<0.001
Total protein, g/L	70.66 ± 6.48	68.89 ± 7.75	<0.001
Globulin, g/L	27.99 ± 4.82	28.34 ± 5.22	0.206
Total bilirubin, μmol/L	14.16 (10.80–18.30)	13.60 (10.23–18.15)	0.153
Direct bilirubin, μmol/L	4.59 (3.48–6.38)	4.61 (3.40–6.50)	0.948
Indirect bilirubin, μmol/L	9.26 (7.23–11.80)	8.78 (6.60–11.81)	0.026
LDH, U/L	186.35 (168.00–215.00)	189.52 (167.42–223.00)	0.138
Total bile acids, μmol/L	4.40 (2.62–7.67)	4.54 (2.52–8.39)	0.814
Metabolic parameters			
Total cholesterol, mmol/L	4.76 (4.00–5.50)	4.50 (3.82–5.22)	<0.001
Triglycerides, mmol/L	1.50 (1.10–1.99)	1.10 (0.81–1.53)	<0.001
Apolipoprotein A1, g/L	1.34 ± 0.30	1.37 ± 0.33	0.083
Apolipoprotein B, g/L	0.91 (0.77–1.07)	0.82 (0.68–0.96)	<0.001
HDL-C, mmol/L	1.10 ± 0.38	1.23 ± 0.39	<0.001
LDL-C, mmol/L	2.74 ± 0.89	2.50 ± 0.84	<0.001
Fasting plasma glucose, mmol/L	5.90 (5.18–8.28)	5.36 (4.89–6.10)	<0.001
Virological characteristics			
HBV DNA, log^10^ IU/mL	3.57 (3.10–4.34)	3.82 (3.19–4.97)	<0.001
HBV DNA ≥1 × 10⁶ IU/mL, n (%)	28 (7.3)	225 (13.5)	0.001

Values are mean ± SD, median (IQR), or n (%), as appropriate.

### 3.2 Clinical heterogeneity within the MAFLD subgroup

Virological characteristics also differed across the four groups. The lowest unadjusted median HBV DNA level was observed in patients with overweight/obesity-related MAFLD (MAFLD-B) [3.52 (3.07–4.22) log10 IU/mL], followed by MAFLD-D [3.66 (3.14–4.46) log10 IU/mL], Non-MAFLD [3.82 (3.19–4.97) log10 IU/mL], and MAFLD-L [3.84 (3.26–5.22) log10 IU/mL] (overall *P* < 0.001). These descriptive differences suggest potential heterogeneity in virological profiles across metabolic phenotypes; adjusted and *post-hoc* analyses were therefore performed to further evaluate these associations (Table 2). *Post-hoc* pairwise comparisons are presented in Supplementary Table S1.

**Table 2:** MAFLD subtype subgroup analysis.

Variable	Non-MAFLD	MAFLD-D	MAFLD-B	MAFLD-L	*P* value
Demographic and anthropometric characteristics					
Age, years	54.35 ± 12.31	54.76 ± 11.51	51.03 ± 10.98	53.36 ± 12.28	0.001
Body mass index, kg/m^2^	24.07 ± 3.43	27.45 ± 3.42	27.88 ± 3.27	22.01 ± 2.55	<0.001
Weight, kg	67.00 (60.00–75.00)	77.00 (70.00–88.00)	79.00 (71.00–89.50)	60.00 (55.75–65.00)	<0.001
Height, cm	167.35 ± 8.04	168.92 ± 9.12	169.61 ± 8.33	167.47 ± 8.55	<0.001
Liver biochemistry					
ALT, U/L	24.12 (16.43–41.09)	30.60 (23.01–49.68)	33.00 (22.95–50.66)	36.85 (24.92–91.83)	<0.001
AST, U/L	23.80 (18.70–34.76)	23.94 (18.77–34.91)	25.00 (20.00–35.50)	26.16 (21.93–47.09)	0.089
GGT, U/L	20.30 (13.23–39.00)	28.98 (20.03–50.06)	25.33 (17.45–43.89)	37.98 (15.60–96.11)	<0.001
ALP, U/L	71.75 (58.00–90.00)	70.50 (60.00–88.41)	67.59 (57.64–86.00)	77.37 (62.25–87.87)	0.259
Albumin, g/L	40.23 ± 5.89	42.44 ± 5.06	42.73 ± 5.00	39.78 ± 4.70	<0.001
Prealbumin, mg/L	221.75 ± 70.68	262.61 ± 53.48	258.40 ± 74.31	236.32 ± 99.41	<0.001
Total protein, g/L	68.89 ± 7.75	71.21 ± 6.63	70.57 ± 6.31	68.14 ± 6.91	<0.001
Globulin, g/L	28.34 ± 5.22	28.51 ± 4.54	27.68 ± 4.89	28.06 ± 5.53	0.302
Total bilirubin, μmol/L	13.60 (10.23–18.15)	13.93 (10.63–17.54)	14.44 (10.91–18.30)	15.79 (10.57–25.65)	0.327
Direct bilirubin, μmol/L	4.61 (3.40–6.50)	4.49 (3.29–6.15)	4.65 (3.56–6.30)	5.33 (3.55–10.39)	0.449
Indirect bilirubin, μmol/L	8.78 (6.60–11.81)	9.02 (7.03–11.71)	9.42 (7.25–11.75)	10.36 (7.48–14.84)	0.107
LDH, U/L	189.52 (167.42–223.00)	191.69 (166.84–214.02)	184.63 (168.02–215.00)	190.27 (169.30–206.88)	0.526
Total bile acids, μmol/L	4.54 (2.52–8.39)	4.65 (3.14–6.82)	4.21 (2.42–7.83)	7.83 (3.48–8.50)	0.228
Metabolic parameters					
Total cholesterol, mmol/L	4.50 (3.82–5.22)	4.79 (4.12–5.53)	4.77 (4.01–5.52)	4.38 (3.54–5.18)	<0.001
Triglycerides, mmol/L	1.10 (0.81–1.53)	1.61 (1.16–2.27)	1.43 (1.07–1.87)	1.48 (0.96–2.40)	<0.001
Apolipoprotein A1, g/L	1.37 ± 0.33	1.38 ± 0.29	1.33 ± 0.31	1.22 ± 0.35	0.051
Apolipoprotein B, g/L	0.82 (0.68–0.96)	0.93 (0.77–1.08)	0.90 (0.77–1.05)	0.87 (0.72–1.04)	<0.001
HDL-C, mmol/L	1.23 ± 0.39	1.14 ± 0.40	1.10 ± 0.36	0.96 ± 0.39	<0.001
LDL-C, mmol/L	2.50 ± 0.84	2.74 ± 0.97	2.76 ± 0.84	2.51 ± 0.86	<0.001
Fasting plasma glucose, mmol/L	5.36 (4.89–6.10)	9.56 (7.50–13.10)	5.43 (5.01–5.97)	5.33 (4.86–6.11)	<0.001
Virological characteristics					
HBV DNA, log^10 ^IU/mL	3.82 (3.19–4.97)	3.66 (3.14–4.46)	3.52 (3.07–4.22)	3.84 (3.26–5.22)	<0.001
HBV DNA ≥1 × 10^6 ^IU/mL, *n* (%)	225 (13.5)	11 (8.2)	14 (6.1)	3 (13.6)	0.006

Subtype comparisons were conducted among Non-MAFLD, MAFLD-D, MAFLD-B, and MAFLD-L.

In phenotype-specific multivariable analyses, only overweight/obesity-related MAFLD (MAFLD-B) remained significantly associated with lower serum HBV DNA compared with non-MAFLD (*β* = −0.337, 95% CI −0.496 to −0.177; *P* < 0.001; BH-adjusted *P* < 0.001). In contrast, the associations for MAFLD-D (*β* = −0.073, 95% CI −0.321–0.174; BH-adjusted *P* = 0.626) and MAFLD-L (*β* = −0.150, 95% CI −0.752–0.452; BH-adjusted *P* = 0.626) were not statistically significant. The estimate for MAFLD-L was imprecise and should be interpreted cautiously because of the small number of complete cases in this subgroup. The complete phenotype-specific regression results for Models 1–3 are presented in Supplementary Table S2.

### 3.3 Independent association between MAFLD and HBV DNA levels

In the full cohort, MAFLD was associated with lower serum HBV DNA levels in the unadjusted analysis (*n* = 2,057; *β* = −0.307, 95% CI −0.437 to −0.176; *P* < 0.001). The revised multivariable analyses included 1,636 participants with complete covariate data after exclusion of records with extreme platelet values. After adjustment for age, sex, body mass index, liver enzymes, metabolic parameters, and HBeAg status, MAFLD remained associated with lower HBV DNA levels (*β* = −0.244, 95% CI −0.384 to −0.104; *P* < 0.001). Further adjustment for FIB-4 did not materially alter the association (*β*=−0.239, 95% CI −0.378 to −0.099; *P* < 0.001). The R^2^ values for Models 1, 2, and 3 were 0.008, 0.267, and 0.268, respectively (Table 3).

**Table 3:** Linear regression analyses of the association between MAFLD and HBV DNA levels.

Model	Exposure	*n*	*β*	95% CI	*P* value	R^2^	Adjusted R^2^
Model 1 (unadjusted)	MAFLD, yes vs. no	2,057	−0.307	−0.437 to −0.176	<0.001	0.008	0.008
Model 2 (adjusted)	MAFLD, yes vs. no	1,636	−0.244	−0.384 to −0.104	<0.001	0.267	0.263
Model 3 (adjusted)	MAFLD, yes vs. no	1,636	−0.239	−0.378 to −0.099	<0.001	0.268	0.263

Model 1 was unadjusted. Model 2 was adjusted for age, sex, body mass index, alanine aminotransferase, aspartate aminotransferase, triglycerides, total cholesterol, fasting plasma glucose, and HBeAg status. Model 3 was additionally adjusted for FIB-4. HBeAg status was classified as negative (<1.0) or positive (≥1.0). Adjusted models were based on patients with complete data for all included covariates. Regression coefficients and 95% confidence intervals were estimated using HC3 heteroskedasticity-robust standard errors.

### 3.4 Co-expression modules associated with HBV replication status

To explore host transcriptional programs associated with HBV replication, WGCNA was conducted in the HBV transcriptomic dataset (Figure 1). Using an appropriate soft-thresholding power to approximate scale-free topology, genes were grouped into distinct co-expression modules based on topological overlap. Module eigengenes were then examined in relation to HBV DNA stratification (none, ≤ 1 × 10^6 ^IU/mL, and > 1 × 10^6 ^IU/mL). Several modules showed associations with HBV replication levels, including modules displaying either positive or negative correlations with HBV DNA burden. Together, these findings suggest that HBV replication is accompanied by coordinated changes in host gene expression programs rather than changes in single genes.

**Figure 1:**
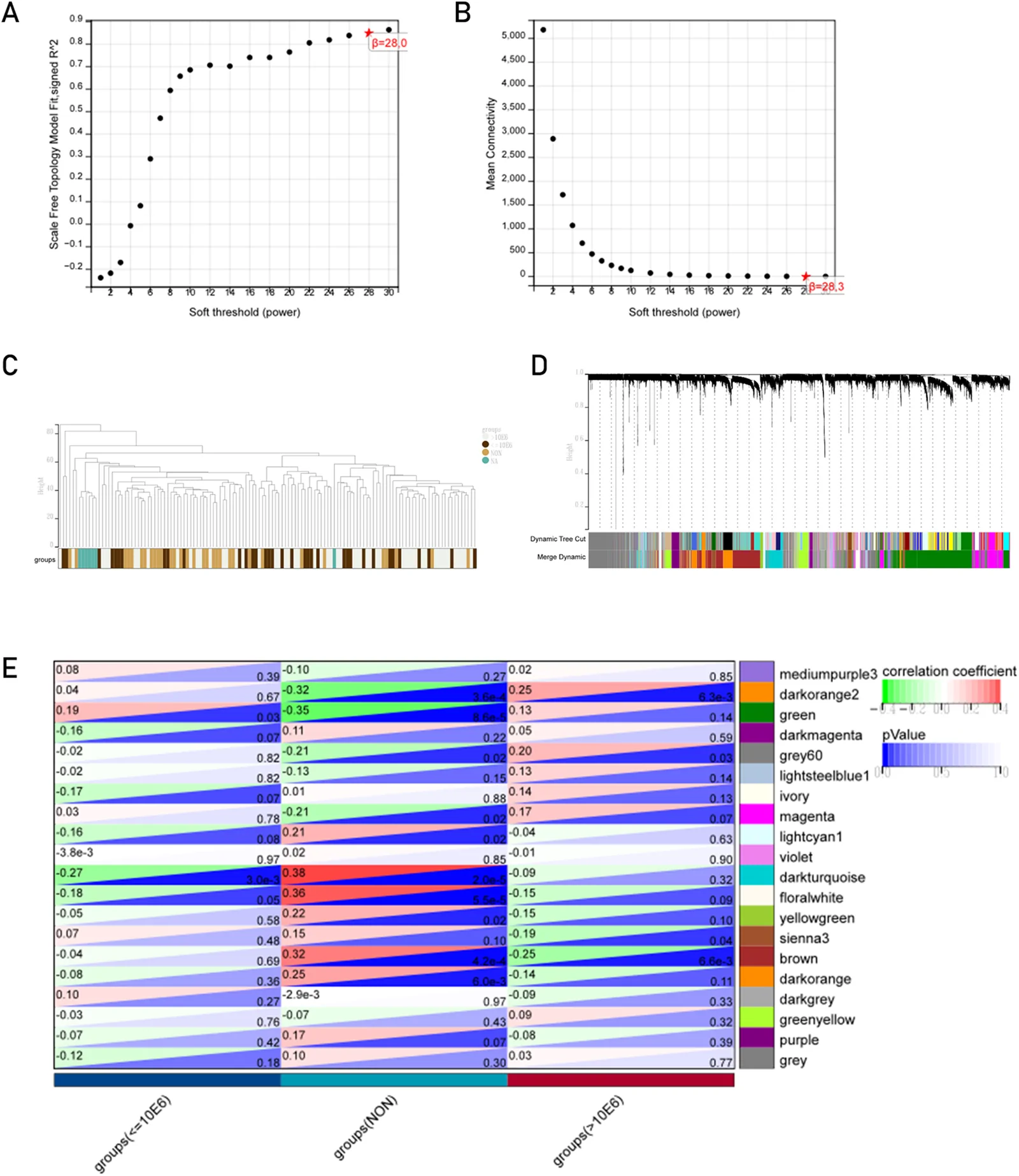
Identification of co-expression modules associated with HBV replication by WGCNA. **(A)** Scale-free topology fit index (signed R^2^) across a range of soft-thresholding powers used for network construction. **(B)** Mean connectivity across the corresponding soft-thresholding powers. **(C)** Hierarchical clustering dendrogram of samples, with the color bar indicating HBV DNA groups. **(D)** Hierarchical clustering dendrogram of genes, with module assignments identified by dynamic tree cutting and subsequent module merging shown by the color bars below the dendrogram. **(E)** Heatmap showing correlations between module eigengenes and HBV DNA groups (≤10^6 ^IU/mL, undetectable, and >10^6 ^IU/mL). Numbers in each cell indicate the correlation coefficient and corresponding *P* value.

### 3.5 Parallel comparison of replication-associated modules across HBV DNA strata

To compare replication-associated modules, eigengene distributions of four selected modules (brown, sienna3, darkorange2, and grey60) were analyzed across HBV DNA stratification groups (Figure 2A). Although multiple modules exhibited changes across replication levels, their patterns differed in both direction and magnitude. To quantify these differences in a standardized way, effect sizes were calculated between patients with high HBV replication (> 1 × 10^6 ^IU/mL) and those with undetectable HBV DNA using Cliff's delta (Figure 2B). Among the four modules examined, the brown module showed the largest effect size, with a consistent inverse directionality relative to HBV DNA burden. In contrast, the remaining modules displayed smaller effect sizes or opposite directional trends. This parallel comparison enabled prioritization of the brown module for subsequent analyses.

**Figure 2:**
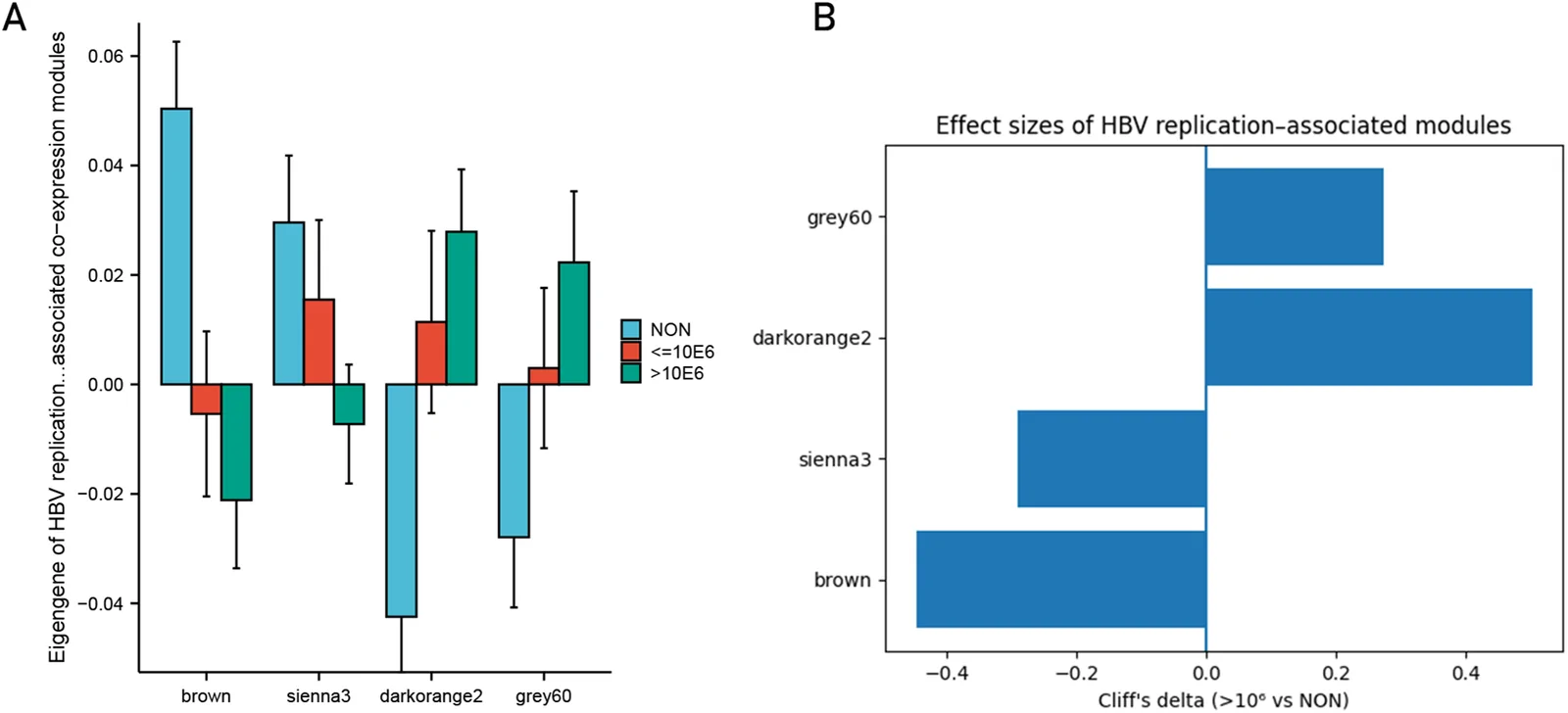
Comparison of HBV replication–associated modules across HBV DNA strata. **(A)** Module eigengene values for selected co-expression modules (brown, sienna3, darkorange2, and grey60) across HBV DNA groups (undetectable, ≤ 1 × 10^6 ^IU/mL, and > 1 × 10^6 ^IU/mL). **(B)** Effect size estimates (Cliff's delta) comparing eigengene values between high HBV DNA (> 1 × 10^6 ^IU/mL) and undetectable groups. The brown module shows the largest effect size and a consistent inverse association with HBV DNA levels.

### 3.6 Cross-cohort characterization of HBV-associated modules in metabolic and fibrotic liver contexts

To assess whether HBV replication–associated transcriptional programs are altered in metabolic liver disease, module gene sets were projected onto independent liver transcriptomic datasets using ssGSEA (Figure 3). In the GSE268360 cohort, module activity scores were compared between normal liver tissues and MAFLD samples. In the GSE240729 cohort, module activity was further examined across fibrosis stages (F0–F2 versus F3–F4). While several modules showed variable changes depending on disease context, the brown module exhibited a consistent alteration across both independent liver datasets. Other replication-associated modules did not display the same degree of concordance between cohorts.

**Figure 3:**
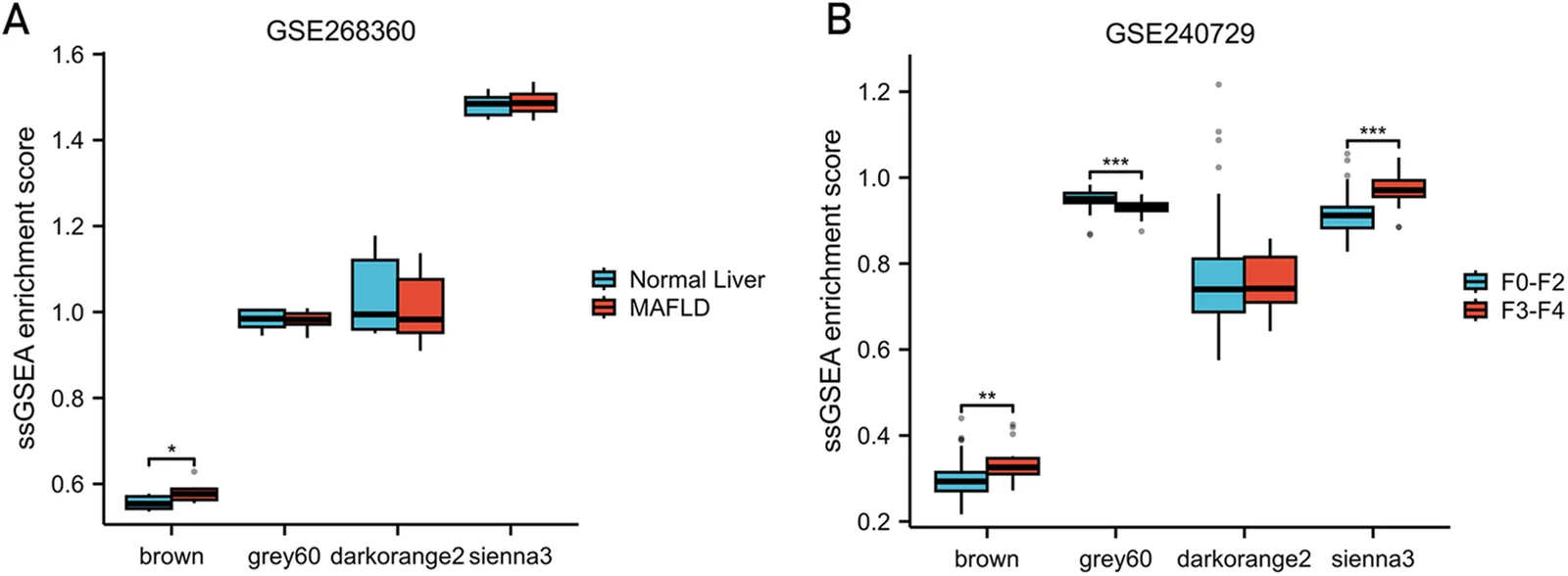
Cross-cohort characterization of HBV replication–associated modules in independent liver transcriptomic datasets. ssGSEA was used to evaluate module activity across independent liver transcriptomic datasets representing distinct metabolic and fibrotic contexts. **(A)** Comparison of module activity between normal liver tissues and MAFLD samples in GSE268360. **(B)** Comparison of module activity between early-stage fibrosis (F0–F2) and advanced fibrosis (F3–F4) in GSE240729. Group differences were assessed using the Wilcoxon rank-sum test.

### 3.7 Functional features of the HBV replication–associated brown module

Functional enrichment analysis of the brown-module signature identified biological processes related to inflammatory regulation, cellular respiration, receptor and vesicular functions, and cellular stress responses. Representative enriched terms included regulation of acute inflammatory response, myeloid leukocyte differentiation, HIF-1 signaling, cellular respiration, oxidoreductase complex, and phospholipid binding (Figure 4).

**Figure 4:**
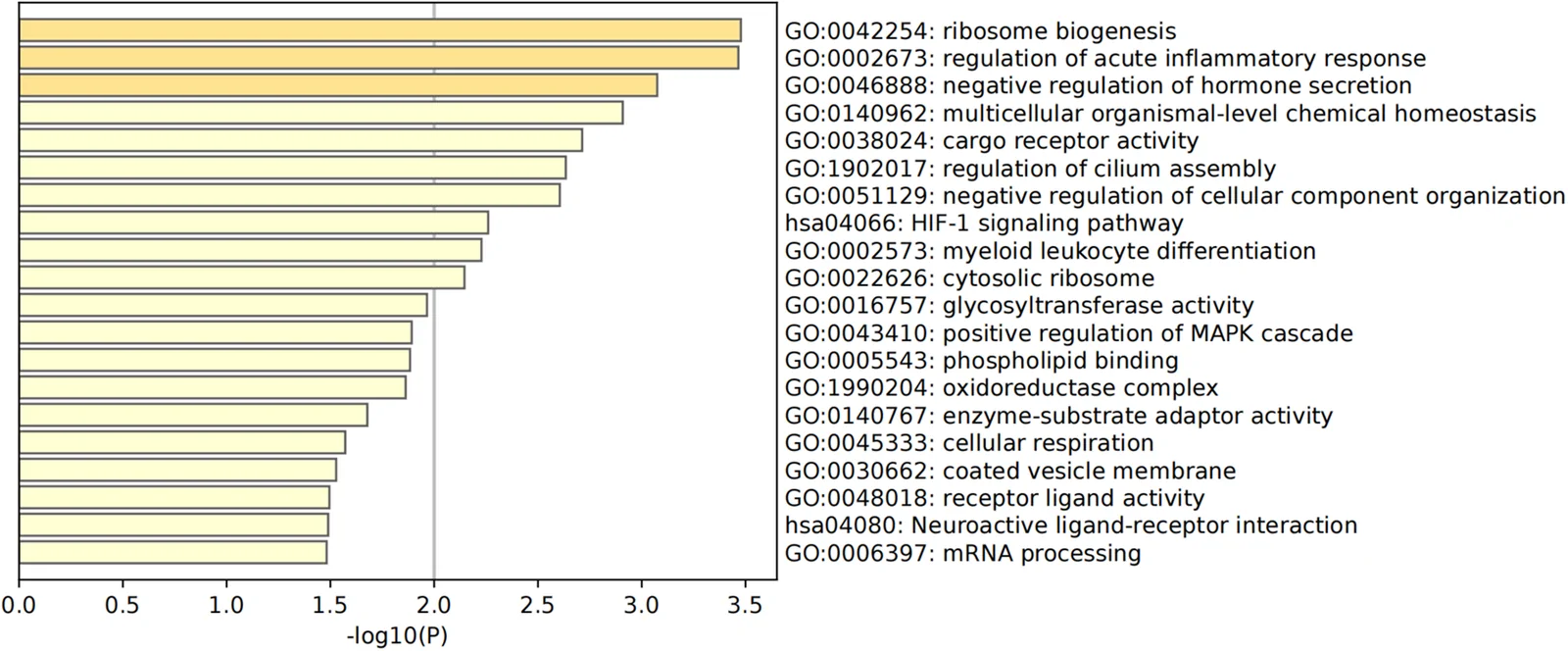
Functional enrichment analysis was performed using Metascape, and representative Gene Ontology and KEGG terms are shown. Bar length represents −log10 (P).

### 3.8 Association between the brown module eigengene and pathway activities in the HBV cohort

Correlation analysis revealed that the brown module eigengene was positively coupled with lipid and cholesterol metabolic signatures, while showing inverse associations with inflammatory and interferon-related pathways in the HBV cohort (Figure 5). These findings are consistent with the hypothesis that HBV replication–associated host programs are metabolically active but relatively dissociated from inflammatory and interferon-driven immune responses.

**Figure 5:**
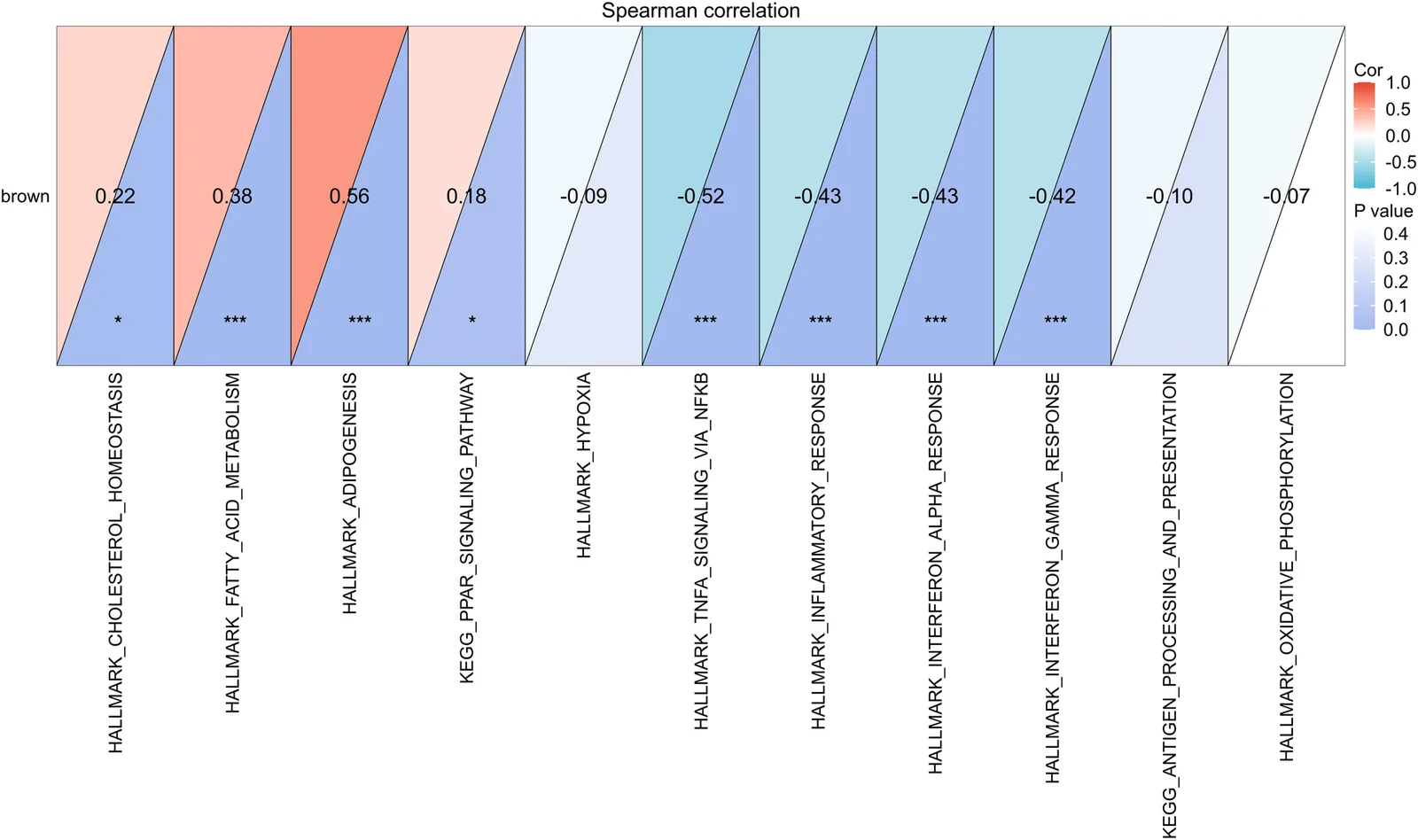
Association between the brown module eigengene and pathway activities in the HBV cohort. Single-sample gene set enrichment analysis (ssGSEA) was used to quantify the activity of selected MSigDB pathways. Spearman correlation coefficients between pathway scores and the brown module eigengene are shown. Pathways were selected to represent lipid and cholesterol metabolism, inflammatory signaling, hypoxia-related stress, and antiviral immune responses. Statistical significance was assessed using Spearman correlation with Benjamini–Hochberg false discovery rate (FDR) correction.

## 4 Discussion

In this large retrospective cohort of patients with chronic hepatitis B, MAFLD was independently associated with lower serum HBV DNA levels, although the association differed across metabolic phenotypes. In phenotype-specific multivariable analyses, MAFLD-B remained associated with lower HBV DNA compared with non-MAFLD after adjustment for demographic, metabolic, biochemical, and virological covariates and correction for multiple testing. In contrast, the associations for MAFLD-D and MAFLD-L were not statistically significant. The estimate for MAFLD-L was imprecise and should be interpreted cautiously because of the small number of patients in this subgroup. These findings suggest heterogeneity in the association between metabolic phenotype and HBV virological activity; however, the observational design does not permit conclusions regarding causality or the direction of these relationships.

The combination of clinical and transcriptomic data suggested that HBV replication was associated with host gene co-expression programs rather than individual genes. The brown module was most consistently negatively correlated with HBV DNA and showed consistent changes when examined across independent liver transcriptomic datasets representing different metabolic and fibrotic contexts. Module annotation pointed to lipid-related processes, membrane organization, and vesicle trafficking, and suggests host lipid-related programs potentially modulate the host intrahepatic environment for optimal HBV replication. Although mechanistically distinct, these pathways may converge toward a cellular environment that is less permissive for efficient HBV replication. Because these datasets were independent, unpaired cohorts with different phenotype definitions, these cross-cohort findings should be considered exploratory and hypothesis-generating rather than direct validation of the clinical MAFLD–HBV DNA association.

Consistent with this finding, lipidomic data provide evidence for the biological plausibility of this link. Chronic HBV infection is associated with systemic lipid compositional changes (decreased hepatotoxic free fatty acids and lysophosphatidylcholines, relative increase in phosphatidylcholine species) that correlate with reduced hepatic injury and steatosis in patients with fatty liver disease (11). Given that HBV assembly and envelopment rely on host membrane integrity, the phospholipid changes are likely to be important, but not decisive, in determining HBV replication capacity (12).

Phospholipid remodeling may also intersect with cellular stress responses relevant to HBV replication. Inhibition of LPCAT3 has been shown to enhance endoplasmic reticulum stress together with increased HBV replication (10). In a separate high-lipid experimental model, SCD1-dependent autophagy was associated with altered HBV replication (13). Prolonged activation or depletion of these stress pathways may affect the capacity of HBV to efficiently use host membrane and secretory pathways. These findings are compatible with, but not definitively supportive of, our network-level observations, and may suggest that induction of autophagy in response to lipid challenges may represent a host-restrictive response in certain metabolic environments.

Immune regulation may represent an additional biological context linking metabolic liver dysfunction with HBV virological activity. In our transcriptomic analysis, the brown module showed inverse relationships with inflammatory and interferon-related pathway activities, suggesting that HBV-associated metabolic transcriptional programs may coexist with alterations in intrahepatic immune signaling. Metabolic liver dysfunction can reshape both innate and adaptive immune responses through changes in hepatocyte stress, lipid-derived signals, cytokine production, and interactions among Kupffer cells, recruited macrophages, NK cells, and T lymphocytes. In chronic HBV infection, interferon signaling, macrophage functional states, NK-cell activity, and HBV-specific T-cell dysfunction are themselves closely related to antiviral immune control (14, 15). Metabolic perturbations may therefore influence HBV virological activity not only through hepatocyte lipid and membrane biology but also through changes in the local immune environment. However, because immune-cell composition and function were not directly measured in our clinical cohort or transcriptomic analyses, these immune mechanisms remain plausible hypotheses rather than mechanisms demonstrated by the present study.

This may be further influenced by metabolic stressors. In experimental models, chronic ethanol synergizes with HBV to enhance the effects of lipid reprogramming and immune dysfunction through HBx-mediated pathways (16). These observations point to the importance of the metabolic milieu in determining HBV replication dynamics.

Overall, our results are compatible with a model in which MAFLD-related metabolic changes, including altered lipid trafficking, membrane phospholipid profiles and ER stress/autophagy pathways, contribute to the permissiveness of the hepatocellular milieu for HBV replication. Different MAFLD phenotypes may have different effects on these pathways, contributing to varied impacts on HBV replication. Taken together, the clinical and transcriptomic findings are compatible with the hypothesis that metabolic and immune characteristics of the hepatic environment may be associated with differences in HBV virological activity. These observations provide biological plausibility but do not establish a causal pathway. Experimental studies using appropriate cellular, animal, or spatial/single-cell approaches will be required to determine whether the identified metabolic and immune programs directly influence HBV replication (17).

## 5 Limitation

Several limitations should be considered. First, the retrospective, single-center observational design precludes conclusions regarding temporal sequence or causality and remains susceptible to residual confounding and selection bias. In particular, systematic information on antiviral treatment and disease duration was not available for all participants. Because nucleos(t)ide analogue therapy is a major determinant of serum HBV DNA levels, unmeasured differences in treatment exposure may have influenced the observed associations despite adjustment for available demographic, biochemical, metabolic, and virological covariates.

Second, hepatic steatosis was identified from imaging examinations obtained during routine clinical care, including ultrasonography, CT, and MRI. Differences in sensitivity among imaging modalities may have introduced misclassification. Direct histological fibrosis staging was also unavailable; FIB-4 was therefore used as a non-invasive surrogate in sensitivity analyses.

Third, the MAFLD phenotype analyses were limited by unequal subgroup sizes, particularly the small MAFLD-L group (*n* = 22 overall and *n* = 15 in the complete-case adjusted analysis), resulting in wide confidence intervals and limited precision. Findings from this subgroup should therefore be regarded as exploratory.

Fourth, the transcriptomic analyses were based on independent public datasets rather than paired liver samples from the clinical cohort. Differences in cohort composition, phenotype definitions, disease stage, and transcriptomic platforms limit direct cross-cohort comparability. Accordingly, these analyses provide complementary biological context but cannot independently validate the clinical association or establish the underlying mechanism. Functional, single-cell, spatial, and experimental studies will be required to validate the metabolic and immune pathways suggested by the present analyses.

Finally, the cohort was derived from a single center in China. Differences in HBV genotype distribution, metabolic characteristics, treatment patterns, and other population-specific factors may limit the generalizability of these findings to other ethnic and geographic populations. External validation in independent, prospectively characterized cohorts is warranted.

## Data Availability

The original contributions presented in the study are included in the article/Supplementary Material, further inquiries can be directed to the corresponding author.
