# Supplementary material for: MAFLD is linked to lower HBV DNA levels in chronic hepatitis B: clinical and transcriptomic insights
Source: Front Med (Lausanne). 2026 Sep 16;13:1926645. doi: 10.3389/fmed.2026.1926645 (PMC13623727; doi:10.3389/fmed.2026.1926645)
Supplement: Supplementary file 1 [file Datasheet1.docx]

MAFLD is linked to lower HBV DNA levels in chronic hepatitis B: clinical and transcriptomic insights

Xiaoyun Gao^1^, Xuan Du^2*^

^1^Department of Geriatric, The Second Affiliated Hospital of Dalian Medical University, Dalian, China

^2^Department of Gastroenterology, The Second Affiliated Hospital of Dalian Medical University, Dalian, China

*** Correspondence:**
Xuan Du
duxuan945@163.com

**Supplementary Table S1. Post-hoc pairwise comparisons among metabolic phenotype groups**

| Variable | Overall test | Non-MAFLD vs MAFLD-D | Non-MAFLD vs MAFLD-B | Non-MAFLD vs MAFLD-L | MAFLD-D vs MAFLD-B | MAFLD-D vs MAFLD-L | | MAFLD-B vs MAFLD-L |
| --- | --- | --- | --- | --- | --- | --- | --- | --- |
| Age, years | 0.001 | 0.982 | <0.001 | 0.981 | 0.024 | | 0.959 | 0.823 |
| Body mass index, kg/m² | <0.001 | <0.001 | <0.001 | 0.025 | 0.647 | | <0.001 | <0.001 |
| Height, cm | <0.001 | 0.139 | <0.001 | 1.000 | 0.864 | | 0.867 | 0.642 |
| Albumin, g/L | <0.001 | <0.001 | <0.001 | 0.984 | 0.967 | | 0.184 | 0.099 |
| Prealbumin, mg/L | <0.001 | <0.001 | <0.001 | 0.770 | 0.947 | | 0.366 | 0.497 |
| Total protein, g/L | <0.001 | 0.003 | 0.008 | 0.966 | 0.863 | | 0.285 | 0.469 |
| Globulin, g/L | 0.302 |  |  |  |  | |  |  |
| Apolipoprotein A1, g/L | 0.051 |  |  |  |  | |  |  |
| HDL-C, mmol/L | <0.001 | 0.081 | <0.001 | 0.010 | 0.705 | | 0.192 | 0.421 |
| LDL-C, mmol/L | <0.001 | 0.011 | <0.001 | 1.000 | 0.998 | | 0.657 | 0.581 |
| Weight, kg | <0.001 | <0.001 | <0.001 | 0.027 | 0.324 | | <0.001 | <0.001 |
| ALT, U/L | <0.001 | <0.001 | <0.001 | 0.019 | 1.000 | | 1.000 | 1.000 |
| AST, U/L | 0.089 |  |  |  |  | |  |  |
| GGT, U/L | <0.001 | <0.001 | <0.001 | 0.129 | 0.427 | | 1.000 | 1.000 |
| ALP, U/L | 0.259 |  |  |  |  | |  |  |
| Total bilirubin, μmol/L | 0.327 |  |  |  |  | |  |  |
| Direct bilirubin, μmol/L | 0.449 |  |  |  |  | |  |  |
| Indirect bilirubin, μmol/L | 0.107 |  |  |  |  | |  |  |
| LDH, U/L | 0.526 |  |  |  |  | |  |  |
| Total bile acids, μmol/L | 0.228 |  |  |  |  | |  |  |
| Total cholesterol, mmol/L | <0.001 | 0.013 | 0.015 | 1.000 | 1.000 | | 0.286 | 0.357 |
| Triglycerides, mmol/L | <0.001 | <0.001 | <0.001 | 0.046 | 0.131 | | 0.805 | 0.902 |
| Apolipoprotein B, g/L | <0.001 | <0.001 | <0.001 | 1.000 | 1.000 | | 1.000 | 1.000 |
| Fasting plasma glucose, mmol/L | <0.001 | <0.001 | 1.000 | 1.000 | <0.001 | | <0.001 | 1.000 |
| HBV DNA, log10 IU/mL | <0.001 | 0.326 | <0.001 | 0.812 | 0.737 | | 0.737 | 0.499 |
| HBV DNA ≥1×10⁶ IU/mL | 0.006 | 0.422 | 0.006 | 1.000 | 1.000 | | 1.000 | 0.708 |

Post-hoc pairwise comparisons were performed for variables with a statistically significant overall test.

**Supplementary Table S2. Associations of MAFLD phenotypes with serum HBV DNA levels**

| Model | Comparison | n | β | 95% CI | P value | BH-adjusted P |
| --- | --- | --- | --- | --- | --- | --- |
| Model 1 | MAFLD-D vs Non-MAFLD | 2057 | −0.228 | −0.438 to −0.018 | 0.033 | 0.050 |
|  | MAFLD-B vs Non-MAFLD | 2057 | −0.386 | −0.541 to −0.230 | <0.001 | <0.001 |
|  | MAFLD-L vs Non-MAFLD | 2057 | 0.041 | −0.530 to 0.612 | 0.888 | 0.888 |
| Model 2 | MAFLD-D vs Non-MAFLD | 1636 | −0.073 | −0.321 to 0.174 | 0.561 | 0.626 |
|  | MAFLD-B vs Non-MAFLD | 1636 | −0.337 | −0.496 to −0.177 | <0.001 | <0.001 |
|  | MAFLD-L vs Non-MAFLD | 1636 | −0.150 | −0.752 to 0.452 | 0.626 | 0.626 |
| Model 3 | MAFLD-D vs Non-MAFLD | 1636 | −0.070 | −0.318 to 0.178 | 0.579 | 0.663 |
|  | MAFLD-B vs Non-MAFLD | 1636 | −0.331 | −0.490 to −0.172 | <0.001 | <0.001 |
|  | MAFLD-L vs Non-MAFLD | 1636 | −0.134 | −0.734 to 0.467 | 0.663 | 0.663 |

Model 1 was unadjusted. Model 2 was adjusted for age, sex, body mass index, alanine aminotransferase, aspartate aminotransferase, triglycerides, total cholesterol, fasting plasma glucose, and HBeAg status. Model 3 was additionally adjusted for FIB-4. Non-MAFLD was used as the reference group. Regression coefficients and 95% confidence intervals were estimated using HC3 heteroskedasticity-robust standard errors. P values for the three prespecified subtype-versus-non-MAFLD comparisons within each model were adjusted using the Benjamini–Hochberg false discovery rate procedure.

**Supplementary Table S3. Distribution of imaging modalities used for hepatic steatosis assessment**

| Imaging modality | Positive findings, n (%) |
| --- | --- |
| Abdominal ultrasonography | 291 (72.9) |
| Elastography | 82 (20.6) |
| Abdominal computed tomography (CT) | 67 (16.8) |
| Chest CT | 61 (15.3) |
| Abdominal magnetic resonance imaging (MRI) | 18 (4.5) |

Imaging modality categories were not mutually exclusive because some patients had positive findings on more than one imaging examination. Multiple imaging modalities (≥2 modalities): 103 (25.8%), including two modalities in 86 patients (21.6%) and three modalities in 17 patients (4.3%).
